# Feasibility, enablers and challenges of using timeliness metrics for household contact tracing and TB preventive therapy in Pakistan

**DOI:** 10.1371/journal.pone.0295580

**Published:** 2023-12-11

**Authors:** Bushra Jamil, Divya Nair, Pruthu Thekkur, Neelofar Laeeq, Anum Adil, Mohammed Khogali, Rony Zachariah, Selma Dar Berger, Srinath Satyanarayana, Ajay M. V. Kumar, Aaron Bochner, Amanda McClelland, Razia Fatima, Anthony D. Harries

**Affiliations:** 1 Department of Medicine, Aga Khan University, Karachi, Sindh, Pakistan; 2 Centre for Operational Research, International Union Against Tuberculosis and Lung Disease (The Union), Paris, France; 3 Institute of Public Health (IPH), College of Medicine and Health Sciences (CMHS), United Arab Emirates University (UAEU), Al Ain, United Arab Emirates; 4 United Nations Children Fund, United Nations Development Programme, World Bank, World Health Organization, Special Programme for Research and Training in Tropical Diseases (TDR), WHO, Geneva, Switzerland; 5 The Union South-East Asia Office, New Delhi, India; 6 Department of Community Medicine, Yenepoya Medical College, Yenepoya (Deemed to be University), University Road, Deralakatte, Mangalore, India; 7 Resolve to Save Lives, New York City, New York, United States of America; 8 Common Management Unit (TB, HIV/AIDS & Malaria), Islamabad, Pakistan; 9 Department of Clinical Research, Faculty of Infectious and Tropical Diseases, London School of Hygiene and Tropical Medicine, London, United Kingdom; Institute of Social and Preventive Medicine, SWITZERLAND

## Abstract

**Introduction:**

Screening household contacts of TB patients and providing TB preventive therapy (TPT) is a key intervention to end the TB epidemic. Global and timely implementation of TPT in household contacts, however, is dismal. We adapted the 7-1-7 timeliness metric designed to evaluate and respond to infectious disease outbreaks or pandemics, and assessed the feasibility, enablers and challenges of implementing this metric for screening and management of household contacts of index patients with bacteriologically-confirmed pulmonary TB in Karachi city, Pakistan.

**Methods:**

We conducted an explanatory mixed methods study with a quantitative component (cohort design) followed by a qualitative component (descriptive design with focus group discussions).

**Results:**

From January-June 2023, 92% of 450 index patients had their household contacts line-listed within seven days of initiating anti-TB treatment (“first 7”). In 84% of 1342 household contacts, screening outcomes were ascertained within one day of line-listing (“next 1”). In 35% of 256 household contacts eligible for further evaluation by a medical officer (aged ≤5 years or with chest symptoms), anti-tuberculosis treatment, TPT or a decision for no drugs was made within seven days of symptom screening (“second 7”). The principal reason for not starting anti-tuberculosis treatment or TPT was failure to consult a medical officer: only 129(50%) of 256 contacts consulted a medical officer. Reasons for poor performance in the “second 7” component included travel costs to see a medical officer, loss of daily earnings and fear of a TB diagnosis. Field staff reported that timeliness metrics motivated them to take prompt action in household contact screening and TPT provision and they suggested these be included in national guidelines.

**Conclusions:**

Field staff found “7-1-7” timeliness metrics to be feasible and useful. Integration of these metrics into national guidelines could improve timeliness of diagnosis, treatment and prevention of TB within households of index patients.

## Introduction

Tuberculosis preventive treatment (TPT) is known to stop progression from tuberculosis (TB) infection to tuberculosis disease and is therefore a critical intervention to reduce TB incidence [1]. TPT is one of the preventive actions envisaged by Pillar 1 (Integrated, patient-centred care and prevention) of the World Health Organization (WHO) End TB Strategy which aims to bring about a 90% reduction in TB incidence by 2035 compared with 2015 [2].

Upholding the WHO End TB targets, governments at the UN High Level Meeting on Tuberculosis (UNHLM) made commitments to scale up TPT to at least 30 million people between 2018 and 2022, including 24 million household contacts of TB patients and 6 million people living with HIV (PLHIV) [3]. However, in the four years since this meeting, only 2.2 million household contacts (9% of target) had been provided with TPT [4]. The slow uptake and scale-up of TPT have been attributed to inadequate priority given to TPT by the programmes, deficiencies in recording and reporting of information on TPT implementation, challenges in undertaking household contact investigation, limitations of available diagnostic assays and TPT regimens [1, 5, 6].

Timely initiation of TPT in household contacts is also crucial for preventing TB disease. Previous studies have shown that 83% of children with TB infection developed the disease within the first 3 months of exposure [7]. A meta-analysis showed that timely provision of TB preventive therapy (TPT) could reduce the risk of developing TB disease by 63% among all children exposed to active pulmonary TB patients and by 85% among those identified with TB infection [8]. However, timeliness in initiation of TPT remains sub-optimal, and studies have reported that household contact screening and initiation of TPT among eligible contacts can take up to two months or longer [9, 10].

In 2021, a 7-1-7 timeliness metric was proposed to facilitate pandemic preparedness: detection of the outbreak within 7 days of emergence; notification to public health authorities within 1 day of detection; and completion of seven early response actions within 7 days of notification [11, 12]. We adapted this metric to the context of screening and management of household contacts of TB patients as follows: *First 7*- household contacts of the index TB patient are line-listed within seven days of the index patient initiating anti-TB treatment; *Next 1*- line-listed household contacts are screened for symptoms suggestive of TB within the next one day; *Second 7*- eligible household contacts are initiated on anti-TB treatment or TPT or a decision is taken to receive no drugs within seven days of symptom screening.

Pakistan, a South Asian country, ranks fifth globally, in terms of TB incidence. Despite this burden, in 2021, only 3% of children under-five years of age who were household contacts of bacteriologically confirmed TB patients were initiated on TPT [4]. In this study, we aimed to assess the feasibility, enablers, challenges and perceptions of utility of implementing the 7-1-7 timeliness metric for screening and management of household contacts of patients with bacteriologically confirmed pulmonary TB in Karachi city, Pakistan.

## Materials and methods

### Study design

The study used an explanatory mixed methods approach. The quantitative component of the study was a cohort study design and the qualitative component was a descriptive design involving focus group discussions.

### Study setting

#### Study sites

The study was conducted in Karachi city of the Sindh Province of Pakistan. Karachi is the most populous city of Pakistan with an estimated population of over 16 million, and a population density of over 24,000 people per square kilometre [13]. The study was conducted in five tertiary care hospitals which cater for 60 percent of the TB case burden in Karachi. In the paper these hospitals are referred to as Facility A, B, C, D and E. All were public hospitals except Facility A which was a private hospital. Each of these hospitals had a dedicated TB care provision center for managing patients with TB, which included contact investigation in accordance with national guidelines [14]. As part of the National TB program, in each of these centers there was a dedicated TB treatment facilitator (known as the “DOTS facilitator”) who registered patents at the time of initiation of treatment, maintained their TB treatment cards and counselled them regarding treatment adherence, cough hygiene, nutrition and follow-up.

Facility A and D were large hospitals with multiple departments where patients could be diagnosed with TB, initiated on treatment and then referred to the TB care provision center. Facility C was an exclusive chest disease hospital with well-established diagnosis and contact tracing procedures. Facility B and E were tertiary hospitals with dedicated chest clinics and onsite TB diagnostic facilities. The Facilities B, C and D were located in areas which were home to communities known to have conservative cultural beliefs and practices. Though diagnosis and medications for TB were provided free of cost, Facilities A and E charged nominal user fees for consultation with doctors.

#### Household contact screening / investigation procedures in Pakistan

The National TB guidelines of Pakistan (2019) recommend household contact tracing in all people with bacteriologically confirmed pulmonary TB (hereafter referred to as index patients). The guidelines recommend that index patients should be counselled regarding the importance of contact screening and requested to bring their household members to the health facility to undergo screening for TB [14]. The accepted generic definition of a household contact is “a person who shared the same enclosed living space for one or more nights or for frequent or extended periods during the day with the index patient during the three months before commencement of the current treatment episode” [14]. The management of contacts is guided mainly by their age group to which they belong: ≤5 years of age or >5 years of age [14].

Household contacts who are ≤5 years of age with symptoms suggestive of TB are evaluated for TB by a medical doctor using clinical assessment, microbiological and/or radiological investigations. If TB is diagnosed, they are initiated on anti-TB treatment. If TB is ruled out, these contacts are initiated on TPT. All household contacts who are ≤5 years without symptoms suggestive of TB do not require investigation and are initiated on TPT [14].

Household contacts who are >5 years of age and have no symptoms are not evaluated any further for TPT and are not eligible for this intervention. Symptomatic household contacts who are >5 years of age are evaluated for TB by a medical doctor and initiated on anti-TB treatment if TB is diagnosed. If TB is ruled out, these contacts are treated for their chest symptoms as per the judgement of the treating doctor. The guidelines do not explicitly recommend TPT in symptomatic household contacts who are >5 years of age [14].

### Study population

#### Quantitative component

The study population included all the line-listed household contacts of bacteriologically confirmed pulmonary TB patients (index patients) registered for treatment in the five selected hospitals. A sample size of 450 index patients was calculated with the assumption that the achievement of the first 7 would be 80% with a relative precision of 5%, 95% confidence and 10% non-response. Considering four contacts per patient in Pakistan, 1,600 contacts were expected which would have given an estimate with a precision of 2% if the achievement of the first 1 was 80%. Assuming 25% of the contacts would be eligible for further evaluation as per the NTP guidelines, there would be 400 contacts eligible for assessment of the second 7 component.

To achieve the sample size of 450 index patients, all index patients initiated on treatment in the selected hospitals between January- June 2023 were enrolled.

#### Qualitative component

Three focus group discussions were conducted with all the five male focal points (one per site) and the two female study coordinators, who were involved in conducting household contact screening and investigation. They were aged between 28–35 years, had at least an educational qualification of graduate level and were residents of Karachi. They were the key on-ground implementers and were the only stakeholder group who would be able to provide an understanding of the enablers and challenges that they encountered during the process of implementing the timeliness metrics. These focus group discussions all took place on 22 June 2023.

### Study procedure

#### Quantitative component

In each of the five study sites, a focal point was appointed to implement the study procedures and was paid for this work out of project funds. Each of the focal points was trained in the study procedures prior to implementation of the study.

The focal point was placed at the TB care provision center in these facilities and extracted the names and contact details of index patients initiated on treatment from the TB patient register on a daily basis. The focal points then interviewed each index patient either during their visit to the facility or over the phone. Based on the interview, focal points prepared a line list of all the household contacts of the index patients (including details of age, sex and relationship to the index patient).

The focal points then attempted to screen each line-listed household contact either over the phone or in-person at home or at the health facility for the presence of any symptoms suggestive of TB (namely cough, fever, night sweats, weight loss and haemoptysis). The preferred approach was to conduct screening in person at home or in the facility, in line with the protocol. At the end of the interview, one of four screening outcomes was assigned to each contact (already on TPT, already on anti-TB treatment, symptomatic or asymptomatic). Symptomatic contacts >5 years of age and all contacts ≤5 years of age were eligible for further evaluation as per the national guidelines.

Symptomatic contacts >5 years of age were counselled to consult a medical doctor at any nearby health facility so that they could be evaluated for TB. Caregivers of contacts ≤5 years of age were also requested to take their children to a nearby health facility for assessing their eligibility for TPT and initiation of TPT. The focal points placed at each health facility helped the contacts to register and consult the medical doctor posted at the TB clinic, once they arrived at the hospital. For each household contact who consulted a medical doctor, the investigations with the results and the interventions (anti-TB treatment, TPT, or a decision not to initiate treatment) were abstracted by the focal points from the medical records at the clinic. Specific dates when each of these activities were conducted were also abstracted.

#### Qualitative component

At the conclusion of the quantitative component of the study, DN and PTK conducted three focus group discussions, each discussion involving all the focal points and study coordinators to document the enablers and challenges associated with i) listing and contacting the index patients and their household contacts, ii) performing symptom screening of household contacts, and iii) getting investigations done, starting TPT or anti-TB treatment or a decision on no treatment. The appropriateness of 7-1-7 targets and the utility of the timeliness metrics in screening and managing household contacts were also explored in the final discussion. Insights from the quantitative analysis helped direct DN and PTK to ask specific questions during focus group discussions to understand why there were gaps in implementation in certain facilities, patient or household contact groups. Since the topic of interest was common to all the focal points and study coordinators who were equally involved in the study and did not involve sensitive information, focus group discussions were preferred over in-depth interviews. The focus group discussions offered the additional advantage of intra group discussions and validation, thus enriching the information and reducing subjectivity. Each discussion lasted for one hour and was audio recorded.

### Data capture and analysis

#### Quantitative component

The focal points recorded the information obtained during the index patient interview, household contact screening and investigations in a near-real time basis on a mobile phone based data capture application called EpiCollect5 and uploaded this information on to the EpiCollect5 server on a daily basis. An in-country study coordinator was appointed to oversee this work, and she supervised the data collection activities at the sites. The team at the Centre for Operational Research, The Union, conducted quality checks on the data on a weekly basis or more frequently if necessary, and communicated with the study coordinator regarding any discrepancies that were detected. The study coordinator in turn worked with the focal points at each site to ensure timely resolution of issues detected during the quality checks.

Data collected in EpiCollect5 was downloaded in comma separated values (.csv) file format and imported into STATA® (version 16.0 Copyright 1985–2019, Stata Statistical Software, College Station, TX: StataCorp LLC). A flow chart was used to describe the achievements and attritions at each step in the process of contact screening and management. The number and proportion of individuals who achieved each ‘7-1-7’ target was calculated as follows and reported along with their 95% confidence intervals (CI):

- First 7 = Number of index patients in whom line-listing of household contacts was done within seven days of treatment initiation out of all index patients enrolled.
- Next 1 = Number of household contacts in whom symptom screening was done within one day of listing out of all household contacts line-listed.
- Second 7 = Number of household contacts in whom a decision or action related to diagnosis and initiation of anti-TB treatment or initiation of TPT was taken within seven days of symptom screening out of all contacts who were eligible for further evaluation (contacts ≤5 years and contacts >5 years with symptoms).

Demographic and facility characteristics associated with achievement of each 7-1-7 target was carried out using log binomial regression. Unadjusted relative risks (RR) with their 95% confidence intervals (CI) were reported as measures of association. A p-value of <0.05 was considered statistically significant.

#### Qualitative component

The transcripts of focus group discussions were prepared in English on the same day of the discussion with the use of notes and audio-recording. Thematic analysis was done by PTK and DN using Atlas-Ti software for deductive coding to identify the enablers and challenges in implementing 7-1-7 metrics for household contact investigation. A third investigator (ADH) reviewed the analysis, and decisions on coding were done in consensus. Similar codes were combined into themes. To ensure that the results reflected the data, the codes/themes were related back to the original data. The findings were reported by using ‘Consolidated Criteria for Reporting Qualitative Research’ guidelines (COREQ) [15].

### Ethics statement

The study was approved by the Ethics Advisory Group, International Union Against Tuberculosis and Lung Disease, Paris, France (EAG 04/2022 on 28th June 2022), the Institutional Review Board Ethics Committee, Common Unit (TB, Malaria and HIV/AIDS), Islamabad, Pakistan (F.No.02-IRB-CMU-2022 on 19^th^ July 2022) and the Ethics Review Committee (ERC) of the Aga Khan University (2022-7786-23085) on 12^th^ October 2022.

As household contact screening and the provision of TPT were already part of NTP activities, the Ethics Committees granted a waiver of informed consent for the index patients and household contacts. However, verbal consent was obtained by the focal points from each index patient and household contact who was included in the study. Written informed consent was obtained from the focal points and study coordinators (all adults) who participated in the focus group discussions.

## Results

### Overview of implementation of “7-1-7”

**Fig 1:**
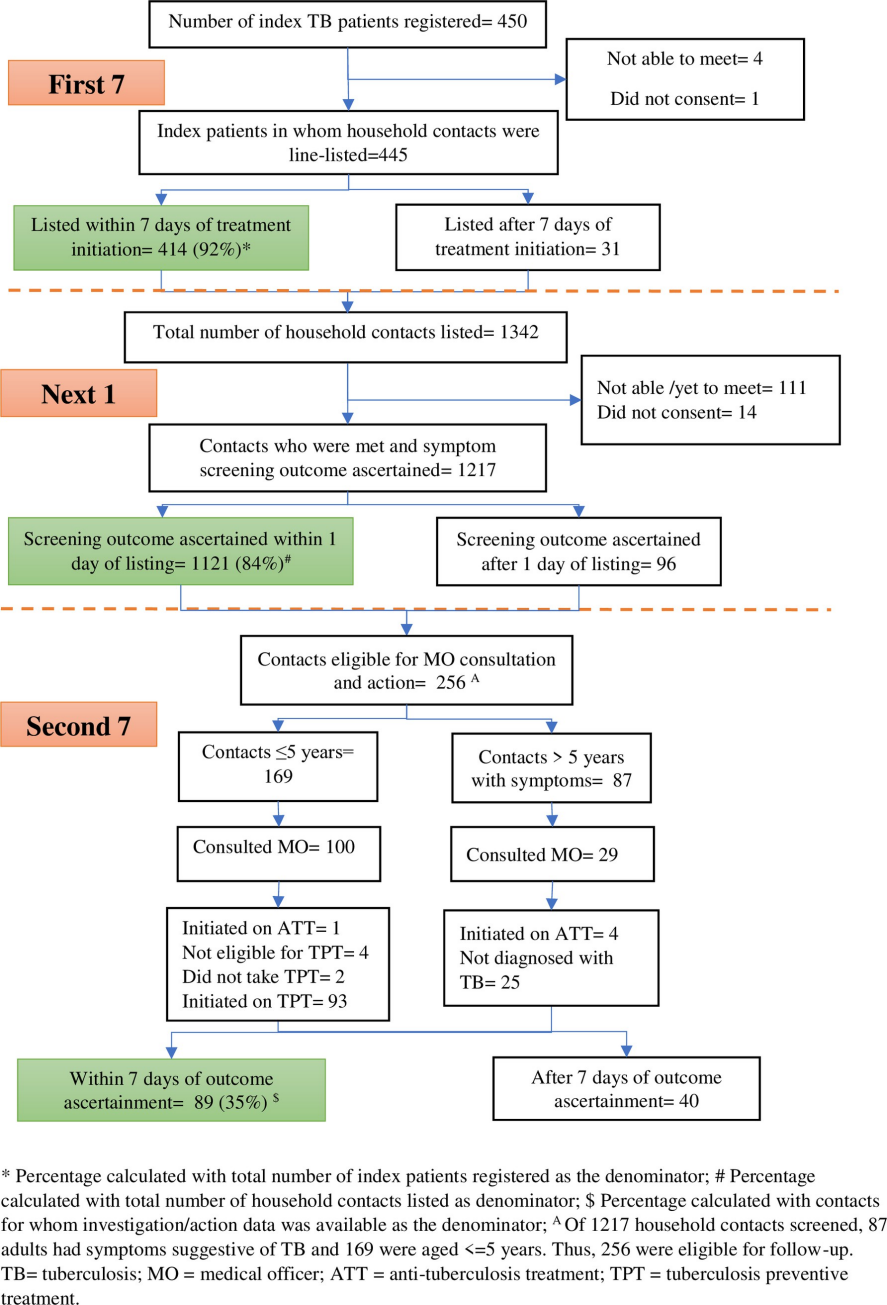
Overview of implementation of 7-1-7 among household contacts of bacteriologically-confirmed pulmonary TB patients treated in healthcare facilities of Sindh province, Pakistan, 15 January-30 June, 2023.

From the five selected health facilities, 450 index patients were recruited during the study reference period. In total, 1342 household contacts were line-listed during interviews with the index patients. The various steps in implementation of household contact screening and TPT provision is shown in Fig 1.

Out of 450 index patients, line-listing of household contacts was done within 7 days of treatment initiation for 414 (92%, 95% CI: 89–94%). Of 1342 household contacts listed, symptom screening outcomes were ascertained within 1 day after line-listing in 1121 (84%, 95% CI:81–85%). Of 256 household contacts eligible for further evaluation by a medical officer to diagnose TB or initiate TPT, the relevant decision or action was taken within 7 days of screening in 89 (35%, 95% CI: 29–41%). The principal reason for not being initiated on anti-tuberculosis treatment or TPT within or outside of seven days was failure to consult a medical officer. Only 129 (50%) of 256 contacts consulted a medical officer: 100 (59%) of 169 contacts ≤ 5 years and 29 (33%) of 87 contacts > 5 years of age.

### Line-listing of household contacts

Characteristics of 450 index patients recruited for the study are shown in Table 1. Of the 450 index patients, the focal points were not able to meet four patients and one patient refused to disclose his/her contacts. In 414 (92%) index patients, the line-listing activity was performed within the first 7 days of their treatment initiation. Index patients ≤14 years had all their household contacts line-listed within seven days, and those who were treated in health facilities B, C and E line-listed their household contacts within seven days in 95%, 98% and 100% of cases respectively. With respect to the reference groups shown in Table 1, those index patients who were aged ≤14 years (RR-1.1, 95% 1.0–1.2) and recruited from health facility B (RR-1.4, 95% 1.2–1.7), health facility C (RR-1.5, 95% 1.2–1.7) and health facility E (RR-1.5, 95% 1.3–1.8) had a relatively higher achievement of the *First 7*.

**Table 1:** Characteristics of index patients with bacteriologically-confirmed PTB initiated on anti-TB treatment in relation to line-listing their household contacts and completing line-listing within the first 7 days in selected health facilities of Sindh province, Pakistan, during January to June 2023.

Characteristics of index patients	Total	Patients whose HHC were line-listed		Patients whose HHC were line-listed within 7 days (“First 7”)		Crude RR (95% CI)^2^	p-value
	N	n	(%)^1^	n	(%)^1^		
**Total**	450	445	(99)	414	(92)	-	-
**Age (in years)**							
≤ 14	24	24	(100)	24	(100)	1.1 (1.0–1.2)	0.03
15–29	196	195	(99)	181	(92)	1.0 (0.9–1.1)	0.84
30–44	97	95	(98)	88	(91)	0.9 (0.9–1.1)	0.86
45–59	74	73	(99)	67	(90)	0.9 (0.9–1.1)	0.84
≥60	59	58	(98)	54	(91)	Ref	
**Gender**							
Male	218	215	(99)	201	(92)	Ref	
Female	231	229	(99)	212	(92)	0.9 (0.9–1.1)	0.87
Other	1	1	(100)	1	(100)	-	-
**Health facility**							
A	29	28	(96)	23	(79)	1.2 (0.9–1.5)	0.18
B	105	101	(96)	100	(95)	1.4 (1.2–1.7)	<0.001
C	130	130	(100)	127	(98)	1.5 (1.2–1.7)	<0.001
D	66	66	(100)	44	(67)	Ref	
E	120	120	(100)	120	(100)	1.5 (1.3–1.8)	<0.001

^1^ Row percentage among total number of index patients approached in each category

^2^ The relative risk (RR) with 95% confidence interval (CI) for achieving the “first 7” milestone with total number of index patients in each category as denominator. Not achieving the “first 7” includes those in whom HHC were never listed and those in whom HHC were listed after 7 days.

PTB = pulmonary tuberculosis, HHC = household contact

The enablers and challenges for line-listing of household contacts within the first 7 days from focus group discussions are shown below.

**The enablers** were:

a) placement of focal points at the TB care provision center in each of the health facilities, which enabled focal points to get in touch with index patients as soon as their treatment was initiated. One of the focal points explaining this said:

“*Our work started from the time that the TB treatment card was opened for a patient*. *I used to sit with the hospital staff who initiated and filled the TB treatment card for patients*. *I was able to extract patient details from the treatment card and talk to patients then and there to confirm the details*. *This was easy and feasible for me*.*”*—Focal point from a facility with a high achievement of ‘*First 7*’

b) support from the DOTS facilitators and hospital management team including treating physicians, who provided timely information about diagnosis and treatment initiation of the index patient.

“*The DOTS facilitator was very helpful and made sure that I was informed whenever there was a new patient*. *The dean of the hospital visited the department and requested all doctors to cooperate with me*.*”*—Focal point from a facility with a high achievement of ‘*First 7*’

**The challenges** were:

a) multiple points of diagnosis and treatment initiation in large multi-disciplinary health facilities, which made it difficult to trace each index patient and contact them in a timely manner.

“*This is a tertiary care hospital dealing with so many patients*, *with multiple departments and doctors*. *Some patients are diagnosed with TB in the Pulmonary Medicine department*, *some in the Infectious Medicine department*, *some in the Intensive care unit*, *some in the Internal Medicine department*, *some in the Gastroenterology department and in our community health clinics*. *Some patients are diagnosed during evaluations as part of research in clinical trial units*. *Treatment initiation can happen at diverse places within the hospital and one field coordinator may not be able to be in all places at once*.*”*- Focal point from a facility with a low achievement of ‘*First 7*’

b) difficulty in establishing contact with index patients, especially those traveling from hard-to-reach areas for the purpose of treatment initiation in facility D, as they did not have access to phones or they resided in hard-to-reach areas.

“*There are some security issues in that area and due to these issues people do not share their phone numbers or contact information”*—Focal point from facility D

“*I was not able to travel to meet patients*, *especially if they were from far-off or rural areas*. *If I was not able to meet the patient on the day that he/she visited the hospital for treatment initiation*, *then I had to wait until their next hospital visit when they obtained medicine refills to talk to them*. *Usually these patients come after one week for the next refill*.*”*—Focal point from facility D

The focal points agreed that seven days might not be required for line-listing household contacts after treatment initiation of the index patient and suggested that a target of three days might be more appropriate.

### Symptom screening of household contacts

Characteristics of the 1342 household contacts who were line-listed are shown in Table 2. Of 1342 household contacts, the focal points were not able to contact 111 (8%) and 14 (1%) did not consent to take part in screening. Of the 1217 household contacts screened, 170 were aged ≤5 years. Of the 1217 screened, 17 (one aged ≤5 years and 16 aged >5 years) were already on anti-tuberculosis treatment and were not screened for symptoms suggestive of TB. Of the 1031 household contacts aged >5 years, 87 (8%) had chest symptoms. Therefore, 256 household contacts were eligible for further evaluation: 169 aged ≤5 years who were not already on anti-tuberculosis treatment and 87 aged >5 years who had chest symptoms.

**Table 2:** Characteristics of household contacts of index patients with bacteriologically-confirmed PTB who were visited by focal points, had their screening outcomes ascertained and completed outcome ascertainment within 1 day in selected health facilities of Sindh province, Pakistan, during January to June 2023.

Characteristics of household contacts	Total	Met by focal points		Screening outcomes ascertained		Screening outcomes ascertained within 1 day (“Next 1”)		Crude RR (95% CI)^2^	p-value
	N	n	(%)^1^	n	(%)^1^	n	(%)^1^		
**Total**	1342	1231	(91)	1217	(91)	1121	(84)		
**HHC age (in years)**									
≤ 5	180	171	(95)	170	(94)	145	(81)	Ref	
6 and above	1162	1060	(91)	1047	(90)	976	(84)	1.0 (0.9–1.1)	0.28
**HHC gender**									
Male	717	674	(94)	666	(93)	603	(84)	1.0 (0.9–1.1)	0.52
Female	622	554	(89)	548	(88)	515	(83)	Ref	
Other	3	3	(100)	3	(100)	3	(100)	1.2 (1.1–1.2)	<0.001
**Relationship with index patient**									
Partner	200	179	(89)	175	(73)	158	(79)	Ref	
Child	469	428	(91)	423	(90)	381	(81)	1.0 (0.9–1.1)	0.51
Parent	228	213	(93)	212	(93)	197	(86)	1.1 (1.0–1.2)	0.04
Siblings	297	272	(92)	268	(90)	255	(86)	1.1 (0.9–1.2)	0.05
Others	148	139	(94)	139	(94)	130	(88)	1.1 (1.0–1.2)	0.03
**Health facility**									
A	84	84	(100)	84	(100)	77	(92)	2.6 (2.3–3.1)	<0.001
B	389	389	(100)	389	(100)	387	(99)	2.9 (2.5–3.2)	<0.001
C	316	210	(66)	196	(62)	110	(35)	Ref	
D	257	257	(100)	257	(100)	257	(100)	2.9 (2.5–3.3)	<0.001
E	296	291	(98)	291	(98)	290	(98)	2.8 (2.4–3.3)	<0.001

^1^ Row percentage calculated among total number of household contacts in each category

^2^ The relative risk (RR) with 95% confidence interval (CI) for achieving the “next 1” milestone with total number of household contacts in each category as denominator. Not achieving the “next 1” includes those HHC in whom screening was never done and those in whom screening was done after 1 day.

PTB = pulmonary tuberculosis; HHC = household contact

Out of 1342 household contacts, the symptom screening outcomes were ascertained within 1 day of line-listing for 1121 (84%). Among the five facilities, ascertainment of symptom screening outcomes within one day ranged from 35% to 100%. There was a significantly higher achievement in the “*Next 1*” among parents (RR-1.1, 95% CI- 1.0–1.2) or others (RR-1.1, 95% CI- 1.0–1.2) of index patients compared to partners of index patients and in health facilities A (RR-2.6, 95% CI- 2.3–3.1), B (RR-2.9, 95% CI- 2.5–3.2), D (RR-2.9, 95% CI- 2.5–3.3) and E (RR-2.8, 95% CI- 2.4–3.3) compared to health facility C.

The enablers and challenges from focus group discussions for screening household contacts within one day of line-listing are shown below.

**The enablers** were:

a) familiarity of some focal points with the community where the patients resided. As focal points belonged to the same community or locality, they found it easier to approach the families of index patients and speak to the contacts. Those focal points who belonged to different regions were not usually welcome in close-knit or tightly guarded communities.

“*I was welcome at their houses because I could speak the local dialects (Sindhi and Baloch)*.*”*—Focal point from facility B

b) the opportunity to screen the household contacts who accompanied the index patient to the health facility.

“*Most times*, *the household member accompanied the patient when he/she attended the hospital for treatment initiation*. *I interviewed/screened those members at the hospital itself*. *For those members who were not present at that time*, *we would enquire about their symptoms from those members who had accompanied the patient*.*”*—Focal point from facility C

**The challenges** were:

a) it was not possible to meet household contacts who were employed or children who were at school during the routine working hours of the focal points. Thus, there were delays in meeting such household contacts.

“*It used to happen that when we called or visited the homes*, *the children would not be at home*. *They would be at school*. *The parents would say that they needed to ask the children if they had any symptoms once they returned from school*. *So*, *there were delays in getting information*.*”*—Focal point from facility B

b) some communities were reluctant to allow home visits by focal points as they were considered new to the area unlike some of the female health workers of the health department whom the community were accustomed to.

“*People are usually apprehensive about welcoming new people into their houses*, *especially in the outskirt areas*, *so we had to mostly screen for symptoms over a phone call……Most projects in these areas appoint female health workers from the same area*. *So*, *it is easy for communication with the local people*. *If outsiders enter these areas with a female health worker*, *they are allowed to enter people’s houses and gather information/data*.*”*—Focal point from facility C

c) cultural beliefs and practices in certain communities prohibited male focal points from interviewing female household contacts.

“*Cultural constraints are such that even within the health facility the males would not allow us to speak to the females*. *Males do not allow female patients to speak even to their doctors*. *The only option I had was to gather as much information as possible from the male contacts who accompanied the patients as caretakers or the males who received our phone calls*. *They told me to ask them whatever I needed to ask and not to talk to the female household members*.*”*—Focal point from facility C

d) lack of familiarity and awareness regarding household contact screening

“*First*, *the process of household contact screening was new to most people in the community*. *There was no process for contact listing and screening in Pakistan*, *so it was difficult to explain the process to people*. *Moreover*, *the disease in question is highly stigmatized*.*”—*Study coordinator

As focal points mostly screened household contacts over the phone, they were generally able to ascertain screening outcomes within one day. However, the focal points felt if they were to meet and screen each household contact in-person at the facility or at home, then they needed more time to be able to this. They therefore suggested modifying the target from one to five days.

### Medical officer consultation and TPT provision

Results are shown in Table 3. Of the 256 household contacts eligible for further evaluation, 129 (50%) consulted a medical officer. Only 100 (59%) of 169 household contacts aged ≤5 years and 29 (33%) of 87 household contacts aged >5 years with chest symptoms consulted a medical officer. Among those who consulted a medical officer, five (one aged ≤5 years and four aged >5 years) were diagnosed with TB and initiated on anti-tuberculosis treatment. The symptomatic household contacts aged >5 years in whom TB was ruled out (n = 83) were treated for their symptoms. Of 99 household contacts aged ≤5 years in whom TB was ruled out, 93 (94%) were initiated on TPT.

**Table 3:** Characteristics of household contacts of index patients with bacteriologically-confirmed PTB with screening outcomes ascertained in whom decisions were made and action taken within 7 days of symptom screening in selected health facilities of Sindh province, Pakistan, during January to June 2023.

Characteristics of household contacts	Total	Decisions and action taken						Decisions and action taken within 7 days (“Second 7”)		Crude RR (95% CI)^2^	p-value
		Total		ATT	TPT	Doctor did not prescribe TPT	Eligible but HHC did not take TPT				
	n	n	(%)^1^	n	n	N	N	n	(%)^1^		
**Total**	256	129	(50)	5	94	22	8	89	(35)		
**Symptom Screening Outcome**											
Children ≤5 years	169	100	(59)	1	93	4	2	68	(40)	1.6 (1.1–2.5)	0.02
Symptomatic contacts aged >5 years	87	29	(33)	4	1	18	6	21	(24)	Ref	
**HHC gender**											
Male	139	75	(54)	2	55	13	5	52	(37)	1.2 (0.8–1.6)	0.33
Female	117	54	(46)	3	39	9	3	37	(32)	Ref	
**Relationship with index patient**											
Partner	20	6	(30)	0	0	4	2	6	(30)	1.4 (0.6–3.6)	0.39
Child	139	84	(60)	2	72	6	4	59	(42)	2.1 (1.1–3.8)	0.02
Parent	14	5	(36)	0	0	4	1	4	(29)	1.4 (0.5–3.8)	0.51
Siblings	44	11	(25)	1	7	2	1	9	(20)	Ref	
Others	23	23	(100)	2	15	6	0	11	(28)	1.4 (0.6–2.9)	0.41
**Health facility**											
A	24	6	(25)	3	2	1	0	5	(21)	4.0 (1.0–15.6)	0.04
B	58	5	(9)	1	1	2	1	3	(5)	Ref	
C	45	48	(87)	1	40	6	1	44	(80)	15.5 (5.1–47.0)	<0.001
D	45	9	(20)	0	0	7	2	4	(9)	1.7 (0.4–7.3)	0.46
E	74	61	(82)	0	51	6	4	33	(45)	8.6 (2.8–26.8)	<0.001

^1^ Row percentage calculated among total number of household contacts whose screening outcomes were ascertained.

^2^ The relative risk (RR) with 95% confidence interval (CI) for achieving the “second 7” milestone with total number of household contacts whose screening outcomes were ascertained in each category as denominator. Not achieving the “second 7” includes those HHC in whom decisions and/or actions were never taken and those in whom decisions and/or actions were taken after 7 days.

PTB = pulmonary tuberculosis; HHC = household contact; ATT = anti-TB treatment; TPT = tuberculosis preventive treatment

Of the 256 household contacts, 89 (35%) were started on anti-TB treatment, TPT or had a decision for no drugs within the seven days of symptom screening. There was a significantly higher achievement in the “*Second 7*” among those aged ≤5 years (RR-1.6, 95% CI- 1.1–2.5) compared to chest symptomatic contacts aged >5 years and in health facilities A (RR-4.0, 95% CI- 1.0–15.6), C (RR-15.5, 95% CI- 5.1–47.0) and E (RR-8.6, 95% CI- 2.8–26.8) compared to health facility B. Health facility C performed the best, achieving 80% of decisions and actions within the second seven days.

The enablers and challenges from focus group discussions for mobilizing contacts for evaluation and taking decisions or actions regarding further care within 7 days are shown below.

**The enablers** were:

a) established systems for contact screening in some facilities

“*They know how to capture household contact details and do their screening*. *Their processes are very well defined*. *The medical officers used to make sure that all symptomatic contacts (children and adults) underwent chest x-rays then and there*.*”*- Focal point from Facility C

b) efforts made to counsel patients and contacts by treating doctors

“*Sometimes household contacts took the disease lightly and did not know that it could spread to the family members*. *After counselling*, *they understood the importance of screening*.*”*—Focal point from Facility B

**The challenges** were:

a) reluctance to visit health facilities in the absence of any financial support for covering travel costs, direct medical costs and fear about losing daily wages.

“*There was an issue with conveyance*. *Many of the contacts received daily wages for work*, *so it was matter of losing their day’s earnings*.*”*- Focal point from Facility D

“*They (household contacts) used to say they stayed far away and could not come to the clinic without any support for conveyance*.*”*—Focal point from Facility C

b) working contacts and school children not being able to take time off for visits to the health facility.

“*They preferred to visit the hospital on their day-off or the children’s school holiday*, *that is Sunday*, *but on those days the hospitals were closed*.*”*- Focal point from Facility B.

c) fear and stigma of being diagnosed with TB if they were evaluated

“*There is intense stigma associated with tuberculosis and people are very scared of being diagnosed with TB*. *This requires a major awareness campaign*. *People are just not aware*, *especially those who reside in the outskirts”*–Study coordinator

d) shortage of TPT drugs.

“*The staff said if they found someone required TPT they would not be able to provide TPT*. *They said that they did not have secure supplies of TPT*. *So*, *they did not encourage screening of contacts–overall- in adults and in children*.*”*- Focal point from Facility B

The focal points felt that the critical step in achieving the ‘second-7’ was mobilizing the household contacts for evaluation. Some contacts were reluctant to be evaluated and never made it to the health facility. However, the focal points suggested that seven days was an appropriate target to complete the evaluation of contacts and make decisions, provided interventions to mobilize contacts for evaluation were put in place.

### Utility of timeliness metrics for household contact screening and management

Overall, the focal points felt that the clear timeliness metrics and corresponding targets that were set for each step of the screening and management process improved their efficiency and enabled them to carry out their tasks in a systematic way. They strongly recommended that such timeliness metrics be integrated within the national TB programme guidelines to optimize household contact screening and management.

“*The mind was set that we had to achieve household contact screening in the set period of time*. *We used to work accordingly and try to complete the steps as soon as possible within that time*. *If we did not have this time metric*, *then we would have worked at our own pace*. *Things might have been delayed*.*”*- Focal point for Facility B

**Fig 2:**
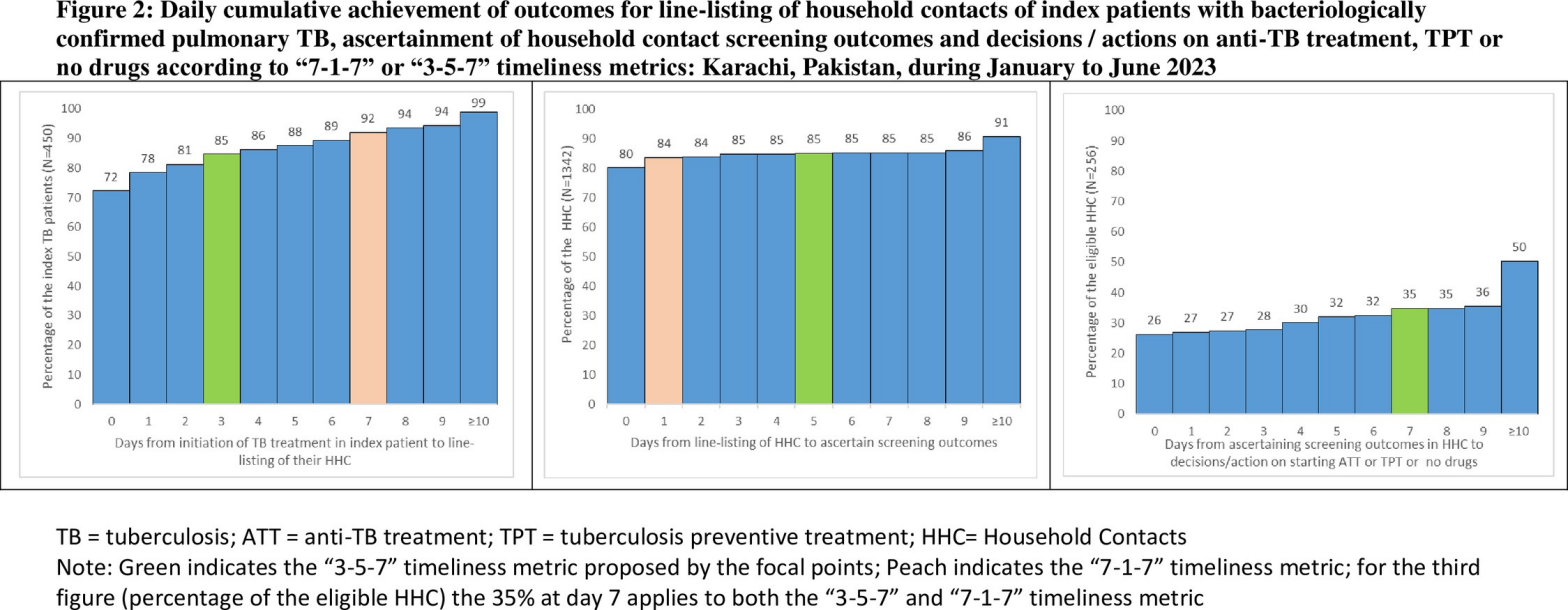
Daily cumulative achievement of outcomes for line-listing of household contacts of index patients with bacteriologically confirmed pulmonary TB, ascertainment of household contact screening outcomes and decisions / actions on anti-TB treatment, TPT or no drugs according to “7-1-7” or”3-5-7” timeliness metrics: Karachi, Pakistan, during January to June 2023.

However, the focal points suggested that an alternative timeliness metric of 3-5-7 be used instead of 7-1-7. In the current cohort, a 3-5-7 metric would have resulted in listing of contacts of 85% of index patients within three days of treatment initiation, symptom screening of 85% of household contacts household within five days of listing and actions/decisions on TPT within seven days of screening in 35% of eligible household contacts as shown in Fig 2.

## Discussion

This study demonstrates that a timeliness metric, similar to that proposed in 2021 to respond to pandemics and public health threats [12], can be applied to the screening and management of household contacts of index patients with bacteriologically confirmed PTB.

For the first two components of “7-1-7”, the overall performance of achieving the timeliness metrics of “seven days” and “one day” was satisfactory at 92% and 84% respectively. Some facilities performed exceptionally well, with three achieving between 95–100% for the “*First seven*” and three achieving between 98–100% for the “*Next one”*. The second seven timeliness metric, however, proved problematic with an overall performance of 35%, although one health facility managed to undertake decisions and actions in 80% of household contacts within the seven days. The poor performance in this last component was largely due to challenges faced by household contacts in consulting a medical officer. This occurred in over half of the contacts eligible for intervention.

The focus group discussions with the focal points of the health facilities provided useful insights into the enablers and challenges of implementing “7-1-7”. The key enablers for the first two components were having focal points embedded within the national TB programme structure and having focal points who were well known and trusted in the catchment community. It was also relatively easy to screen the household contacts who accompanied the index patients when the latter group came to the facility for registration and treatment. The key challenges were tracking down index patients from the multiple diagnostic and treatment points in large multi-disciplinary health facilities (A and D) and the difficulties in contacting index patients and their contacts at home, especially for those who were employed and who were school-aged children. Other practical considerations included the reluctance of households with female contacts to be visited and interviewed by male focal points, especially in communities known to have conservative cultural beliefs and practices (as observed in facilities B, C and E). These problems are not confined to Pakistan and have been found elsewhere. Household contacts are not always present for home visits, requiring repeat visits on other days [16], and other researchers have found that conducting symptom screening and symptom screening outcomes are better when household contacts are parents and in certain TB units compared with others [17].

For the second 7 component, counselling by treating doctors regarding the importance of household screening was considered to be an important enabling factor. However, recurring themes about cost of travel, direct medical expenses (facilities A and E), loss of daily earnings while attending health facilities, fear of being diagnosed with TB and shortages of TPT drugs in the facilities to take preventive medication are important and they are established challenges for implementing TPT in household contacts [18–20]. These issues must be addressed if progress is to be made, not only in Pakistan but in many other countries as well.

Despite the challenges of achieving the second 7 component, over half of the contacts ≤5years eligible for TPT were initiated on it, including a substantial proportion within seven days, and this uptake in our study was better than what has been found in previous observational studies in Africa and Asia [17, 21–23]. When comparing with other settings, however, interpretation needs to be cautious as the guidelines in Pakistan recommend further evaluation only among household contacts <5 years of age and symptomatic household contacts aged >5 years.

The study had several strengths. It was conducted within the routine health care setting in Sindh province, Pakistan, and the data were quality checked on a weekly basis and more frequently if needed. The quantitative findings guided the qualitative discussions and a combination of the two techniques provided a better understanding of the enablers and barriers in implementing the 7-1-7 metric. The conduct and reporting of the quantitative and qualitative components of the study followed STROBE and COREQ guidelines respectively [15, 24].

The main limitations were the lack of a concurrent or historical control group to compare TPT uptake in those eligible for this intervention and inability to conduct qualitative interviews among index patients and their household contacts due to funding constraints. The achievements of the “*First 7*” and “*Next 1*” were slightly underestimated as those who did not provide informed consent were considered to have failed the timeliness metrics, irrespective of receipt of services. In addition, 7-1-7 was implemented by focal points appointed, trained and supported for this study. Whether this could be done within the routine program settings using existing programmatic staff is not clear.

Household contacts of TB patients, especially infants and young children, are at high risk of contracting TB [25–27]. As explained earlier, TPT considerably reduces this risk with protective effects lasting for several years after a course of preventive therapy [28, 29]. Based on this evidence, WHO recommends systematic screening of household contacts for TB and administration of TPT after excluding active TB [1, 30].

The 7-1-7 framework described in this paper provides a workable structure to diagnose, treat and prevent TB within this high-risk environment, and, crucially, introduces a timeliness metric into the intervention so that action is taken sooner rather than later to halt TB transmission. The framework clearly shows that while line-listing and screening work well, the opportunities to administer TPT are not taken up. More needs to be done to educate and apprise household contacts of the risk of TB and the benefits and safety of TPT, so that they are less reluctant to undergo contact screening and take TPT.

The financial constraints faced by contacts was a major challenge which prevented them from travelling to a clinic for screening and further evaluation. However, the programmatic guidelines recommend that contacts be asked to visit the TB care facilities for screening, and without a solution to this conundrum it will be challenging to expand TPT in Pakistan. Provision of social support to households to cover costs of travel and loss of daily earnings as well as strengthening outreach services through the use of mobile vans, point of care diagnostics and skilled staff for diagnosis, treatment and prevention should be considered. The effectiveness of these strategies in improving screening and TPT initiation should also be assessed within the local context before they are implemented on a large scale.

The focal points suggested that a 3-5-7 timeliness metric would be a better alternative to the 7-1-7 metric, the latter being originally designed for a public health response to outbreaks. Even with the 3-5-7 metric, the steps in household contact management would be completed within the 15-day timeframe.

The suggested changes in the metrics for the first two steps seem rational within the context of programmatic implementation of household contact screening. The 3-5-7 metric would result in listing of household contacts of 85% of index patients within 3 days of treatment initiation (compared to 92% within 7 days). Although there appears to be a decline in achievement, the first step of listing household contacts by interviewing index patients can be achieved in a shorter span of time since this activity is usually performed while the patients visit the hospital for collecting their anti-TB medications. The four days gained by restricting the first step to three days can be utilised for the second step. The second step requires each household contact to be contacted individually and screened for symptoms. It is unlikely that all household contacts would be available for interview within one day of listing; hence, the suggestion of five days for this activity. The last component can stay the same.

In thinking and moving ahead, we need to seriously consider the suggestion from focal points about integrating timeliness metrics into the national TB guidelines as this was found to be highly motivational in implementing contact screening and TPT provision. However, we need to assess whether the focal point suggestion of a 3-5-7 timeliness metric works better than 7-1-7 in Pakistan. This merits further operational research. We also need to determine whether timeliness metrics can be implemented within the programmatic setting by established staff.

## Conclusions

The aim of the study was to assess the feasibility, enablers, challenges and utility of implementing a 7-1-7 timeliness metric for screening and management of household contacts of patients with bacteriologically confirmed pulmonary TB in Karachi city, Pakistan. The 7-1-7 timeliness metrics were found to be feasible to implement and were perceived to improve the efficiency of field staff in conducting household contact screening and management. Furthermore, it was strongly suggested that timeliness metrics be integrated within national TB guidelines which would require operational research to optimize its implementation.
